# Treating and triggering hyperinflammation: tackling hemophagocytic lymphohistiocytosis and HLH-like syndromes in the pediatric cell therapy and critical care setting

**DOI:** 10.3389/fonc.2025.1631557

**Published:** 2025-09-19

**Authors:** Holly Wobma, Lauren A. Henderson, Christine N. Duncan, Susan E. Prockop, Adrienne G. Randolph, Barbara A. Degar, Leslie E. Lehmann, Susanne H. C. Baumeister

**Affiliations:** ^1^ Harvard Medical School, Boston, MA, United States; ^2^ Division of Immunology, Boston Children’s Hospital, Boston, MA, United States; ^3^ Division of Hematology-Oncology, Boston Children’s Hospital, Boston, MA, United States; ^4^ Department of Pediatric Oncology, Dana-Farber Cancer Institute, Boston, MA, United States; ^5^ Division of Critical Care, Boston Children’s Hospital, Boston, MA, United States

**Keywords:** hemophagocytic lymphohistiocytosis, primary HLH, macrophage activation syndrome (MAS), systemic juvenile idiopathic arthritis (sJIA), CAEBV, immune effector cell associated HLH-like syndrome (IEC-HS), pediatric critical care, allogeneic hematopoietic cell transplant

## Abstract

Hemophagocytic lymphohistiocytosis (HLH) describes a severe, hyperinflammatory syndrome that can originate from diverse etiologies, often requiring critical care level management. Primary HLH, initially described in the 1940s, derives from genetic defects that result in uncontrolled immune activation. Although chemotherapy and immunosuppressive agents can temporarily quell inflammation, allogeneic hematopoietic cell transplantation (HCT) is the only curative option. In 2025, HCT is indicated for primary HLH and some etiologies of secondary HLH but remains challenging due to both disease and transplant-related inflammation. Additionally, new cellular therapy approaches to treat malignancy, such as chimeric antigen receptor T cells, can trigger a spectrum of hyperinflammatory complications. Herein, we review the pathophysiology, diagnosis, and evolving management approaches of primary and secondary HLH, ultimately informing our management of hyperinflammation in the setting of new cell therapies.

## Introduction

Inflammation is the natural response to injury or infection. It also requires tight regulation. When there is an imbalance between the level of stimulus and the magnitude and duration of response, hyperinflammation ensues. The quintessential hyperinflammatory disorder is hemophagocytic lymphohistiocytosis (HLH). HLH describes a clinical syndrome of fever, cytopenias, hepatosplenomegaly, hyperferritinemia and organ dysfunction, which is often life threatening, requiring critical care management (1). Key HLH pathophysiology involves dysregulated T cells, natural killer (NK) cells, and macrophage/myeloid cells, resulting in excessive cytokine production.

In healthy individuals, activation of antigen presenting cells (APCs) via pattern recognition receptors facilitates antigen-presentation, production of proinflammatory cytokines, and recruitment and activation of cytotoxic CD8+ T-lymphocytes and NK cells. CD8+ T cells and NK cells proliferate and release inflammatory cytokines such as interferon gamma (IFN-γ) (2, 3). This inflammatory milieu further increases the antigen presentation capacity of APCs and stimulates innate immune cells. One of the mechanisms by which CD8+ T cells and NK cells eliminate infected, autoreactive, or malignant cells, is granule-mediated cytotoxicity. This process involves delivery of cytotoxic granule contents into the immune synapse and perforin facilitated entry into the target cell, inducing target cell apoptosis. Once a successful immune response has been orchestrated and target cells are eliminated, the immune response is downregulated in a controlled fashion. CD8+ T cells eliminate activated APCs, NK cells can induce apoptosis of activated APCs and T cells, and ultimately, most involved immune cells undergo a programmed cell death, with only a small number of memory T cells remaining. When this process becomes dysregulated, there is overproduction of IFN-γ, a central cytokine in HLH pathophysiology.

HLH is separated into ‘primary’ and ‘secondary’ forms, although as discussed below, is often multifactorial. Primary HLH derives from pathogenic genetic variants in granule-mediated cytotoxicity or inflammasome regulation that result in an escalating hyperinflammatory feedback loop and ineffective contraction of the immune response. Granule-mediated cytotoxicity defects result in prolonged synapse time, overproduction of cytokines, accumulation of APCs to stimulate T cell inflammation and defective APC and T-cell clearance. In inflammasome regulation defects, this inflammatory cycle is triggered by excessive production of inflammasome-dependent cytokines.

**Figure 1:**
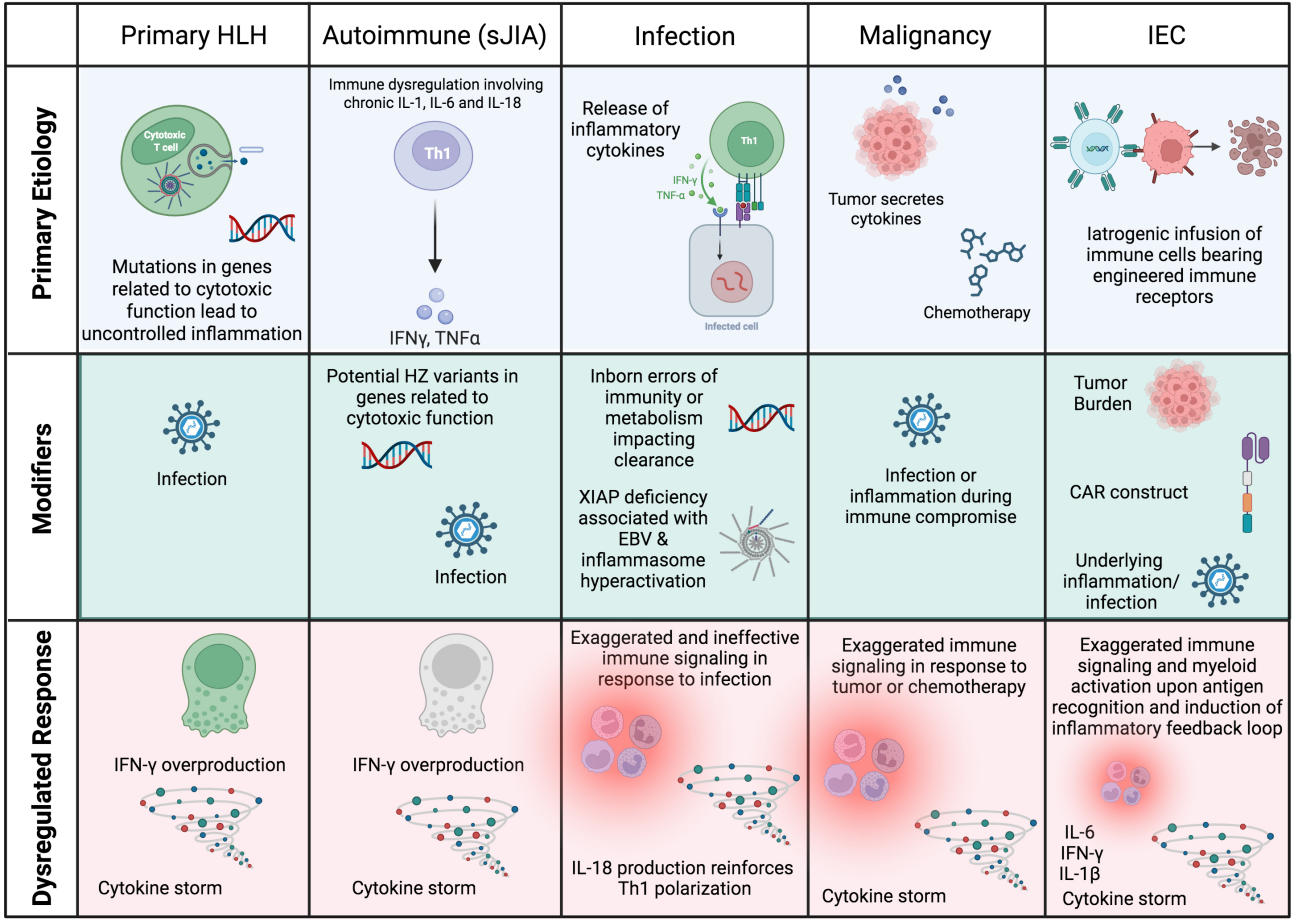
Schematic of the root causes of HLH and HLH-like syndromes in different settings. In most cases, HLH is multifactorial, with a predominating underlying cause that manifests as HLH when additional modifiers or ‘hits’ converge to result in a dysregulated immune response. CAR, chimeric antigen receptor; HSCs, hematopoietic stem cells; HZ, heterozygous; IEC, Immune Effector Cell. Created in BioRender.

In secondary HLH, a predominant underlying etiology (e.g., autoimmune disease, malignancy, infection, administration of Immune Effector Cells (IEC)) in the setting of additional modifiers (e.g., infection, genetic variants) may yield similar functional defects in immune regulation. This model, in which multiple factors converge to surpass a ‘threshold’ for development of HLH, is schematized in **Figure 1** (4). Shared terminal pathophysiology may be marked by the presence of activated, Th1 polarized HLADR+CD38^hi^ CD8+ T cells (5–7), which (amongst other lymphocytes) are the producers of IFN-γ that is characteristic of HLH-related inflammation (8).

Medical management targeting cytokine production and hyperactive immune cells may quell HLH and prevent its recurrence. However, when there is a strong genetic predisposition and/or a chronic underlying disorder, allogeneic hematopoietic cell transplantation (HCT) may be required for definitive treatment. The longstanding experience of cell therapy physicians in HLH management has been instrumental. Additionally, given the illness acuity of patients with HLH and HLH-like syndromes, physicians caring for these patients almost always include those in critical care medicine. Intensivists must balance treating multi-organ dysfunction and immune dysregulation in the context of a patient’s primary disorder. This results in overlapping but unique multidisciplinary management considerations in collaboration with HCT/Cellular therapy, Oncology, Neurology, Rheumatology, Immunology, and Infectious Disease specialists. Here we describe the different causes of HLH and HLH-like syndromes, as well as the role of medical management and HCT.

## Primary HLH

### Classification and pathophysiology

Primary HLH was initially described in the mid-1900s in a series of case reports (9). Infants would develop fevers, cytopenias, hepatosplenomegaly, and neurological features, and within weeks, succumb to disease. Post-mortem bone marrow analysis showed the characteristic finding of ‘hemophagocytosis’ – macrophages (histiocytes) phagocytosing blood cells. This syndrome eventually became known as HLH. The Histiocyte Society initially developed criteria for the diagnosis of HLH in children based on clinical and laboratory criteria for the HLH-94 trial (10). These criteria were subsequently refined for the HLH-2004 trial to include molecular and/or clinical criteria (11). Most recently, revised criteria were proposed in which functional assays in the setting of consistent clinical features can also be used to diagnose HLH (**Table 1**) (12). Additional laboratory tests may support the diagnosis of HLH and be useful for disease activity monitoring. These include detection of the IFN-γ induced protein CXCL9 (since IFN-γ itself is difficult to detect in the bloodstream) (8, 13) as well as HLADR+CD38^hi^ CD8+ T cells, although the latter is not available as a certified laboratory test.

**Table 1:** 2024 revised HLH criteria (12).

To establish a diagnosis of HLH, 1 of 3 criteria must be fulfilled
1. Molecular diagnosis consistent with familial HLH (see in **Table 2**) in a patient with signs/symptoms suggestive of HLH
2. Functional cellular findings consistent with familial HLH in a patient with signs/symptoms suggestive of HLH (such as low or absent NK-cell activity or CD107a Degranulation assay defects)
3. At least 5 of 7 of the below features a. Fever ≥ 38.5°C b. Splenomegaly (≥ 2 cm below the costal margin) c. Cytopenias affecting at least 2 lineages (Hgb <9 g/dL*, platelets <100,000/*μ*L, ANC <1000/*μ*L d. Hypertriglyceridemia (fasting ≥ 3mmol/L) and/or hypofibrinogenemia (≤ 1.5 g/L) e. Hemophagocytosis f. Ferritin ≥500 *μ*g/L g. Soluble IL2R (a.k.a. CD25) ≥2400 U/mL

*<10 g/dL in infants under 4 weeks old.

Primary HLH results from inborn errors of immunity (IEIs) and is estimated to describe 12% of cases of HLH in pediatrics (14). In 1999, perforin (*PRF1*) mutations were found to cause HLH in a subset of patients (15). Perforin is key to the cytotoxic function of CD8+ T cells and NK cells, enabling them to appropriately contract an inflammatory response. Soon thereafter, mutations in other genes (*STX11, STXBP2, UNC13D*) with related functions in the cytotoxic response were implicated in HLH, resulting in a nomenclature of familial HLH (FHL), where ‘familial’ implied inherited, and a corresponding number implied a specific genetic defect in granule mediated cytotoxicity (e.g., FHL2 is HLH due to a perforin defect). As genetic variants driving HLH can be *de novo*, and there is now a larger range of pathways implicated, it is now more common to use the term “primary HLH” and to specify the gene mutation (if known) (16) (**Table 2**). For example, several IEIs that do not affect cytotoxic function but instead regulate the inflammasome (e.g. NLRC4, CDC42) have HLH as a common manifestation (17–19). In these disorders, overactive inflammasome signaling drives activation of caspase-1 and its cleavage products IL-1β and IL-18, which can promote a hyperinflammatory cascade (20). Lastly, IEIs that predispose to poor control of Epstein Barr virus (EBV) infection may increase the risk for HLH and will be discussed in a later section. There may also be an overlap, as XIAP deficiency exhibits both altered inflammasome regulation and increased susceptibility to EBV infection (21).

**Table 2:** Primary HLH (based on an underlying genetic defects).

Defective lymphocyte granule-mediated cytotoxicity		
Familial	PRF1	Deficiency in Perforin, which binds to the target cell and forms multimeric pores in the cell membrane through which granzymes enter.
	UNC13D (Munc13-4)	Defect in docking of cytotoxic vesicles at the immune synapse.
	STX11	Defect in fusing cytotoxic vesicle to the cell membrane for release of perforin and granzymes
	STXBP2 (Munc18-2)	Defect in fusing cytotoxic vesicle to the cell membrane for release of perforin and granzymes
Pigment disorders	RAB27A	Griscelli’s syndrome type 2. Defect in docking cytotoxic vesicles at the immune synapse.
	LYST	Chediak-Higashi Syndrome. Defect in the biogenesis of secretory lysosomes.
	AP3B1	Hermansky Pudlak syndrome type 2. Defect in intracellular protein transport, including within lysosomes

Inflammasome and other dysregulation of cytotoxicity		
XLP-1	SH2D1A (SAP)	Defect in 2B4-mediated cytotoxicity and in T-cell restimulation-induced cell death. Commonly triggered by EBV. X-linked.
XLP-2	XIAP	Dysregulated NLRP3 inflammasome function. Increased effector cell susceptibility to cell death. Commonly triggered by EBV. X-linked (21).
	CDC42	NOCARH syndrome. Defect in the formation of actin-based structures, proliferation, migration, and cytotoxicity. Dysregulated NLRP3 inflammasome function (increased IL-1β and IL-18 production). Increased effector cell susceptibility to cell death (18).
	NLRC4	Constitutively active NLRC4 inflammasome function and increased IL-18 production (19).
EBV susceptibility disorders	MAGT1	Defective Mg^2+^ transporter, Low NKG2D-receptor expression. Defect in cytotoxicity. X-linked (128).
	ITK	Defect in tyrosine kinase function, T cell expansion, and cytolytic capacity. Decrease in invariant NKT cells
	CD27	Lack of CD27 causes defect in T-cell costimulation via CD27 (expressed on T cells) via CD70 (expressed on EBV-infected B cells) (129).
	CD70	Lack of CD70 expression on EBV-infected B-cell and thus costimulation through CD27 for expansion and cytotoxicity of T cells against EBV-infected B-cells (129).
	CTPS1	Deficiency in CTPS1, an enzyme involved in a critical precursors of nucleic acid metabolism leads to impaired T-cell proliferation (130).
	RASGRP1	Lack in RAS activation and therefore MAPK pathway activation resulting in defects in T-cell activation, proliferation, migration and cytotoxicity (131).

Inborn errors of immunity (IEIs) and inborn errors of metabolism (IEM) which may be complicated by HLH		
IEI	SCID, CIDs, DiGeorge syndrome, Wiskott-Aldrich syndrome, Ataxia telangiectasia, Dyskeratosis congenita, ORAI-1 deficiency, CGD, X-linked Agammaglobulinemia, ALPS, STAT1 gain of function, CTLA-4 deficiency, GATA-2 deficiency, TRAPS, FMF, NEMO, TIM3, DOCK8, STAT2, STAT3, PIK3CD (132).	
IEM	Lysinuric protein intolerance (*SLC7A7*), Multiple sulfate deficiency, Biotinidase deficiency, Lysosomal acid lipase/deficiency (Wolman disease), Methylmalonic acidemia, Galactosemia, Gaucher disease, Pearson syndrome, Galactosialidosis, Propionic acidemia, Cobalamin C disease, Niemann-Pick disease, LCHAD deficiency.	
CDG	COG6 (congenital disorder of glycosylation)	

ALPS, autoimmune lymphoproliferative syndrome, CID, combined immune deficiency, SCID, severe combined immune deficiency.

### Medical treatment of primary HLH

Early attempts to treat classic ‘familial’ HLH included adrenocorticotropic hormone, splenectomy, exchange transfusion, and steroids (1). There was also use of chemotherapeutic agents. One agent in particular – etoposide – was selected due to its use in monocytic leukemias suggesting a potential ability to subdue macrophage activation. Indeed, early studies with etoposide were highly promising (22). Ironically, the mechanism of its benefit was different than predicted. Etoposide is a topoisomerase II inhibitor, blocking repair of double stranded DNA breaks, triggering apoptotic cell death. This was shown to be true of activated, cytotoxic CD8+ T cells in murine models of HLH (23). Thus, etoposide eliminates a hyperactive cell population in HLH that would have otherwise been eliminated had there not been a genetic defect in cytotoxic function. In addition to etoposide, intrathecal methotrexate (anti-metabolite) was added to regimens for central nervous system (CNS) coverage, as there is CNS involvement in 30-73% of patients with primary HLH (24). As dexamethasone has better CNS penetrance than other forms of corticosteroids, it emerged as the steroid of choice. Calcineurin inhibitors – particularly cyclosporine – were added to protocols to block NFAT-mediated production of cytokines such as IL-2, TNF-α, and IFN-γ. Finally, allogeneic HCT was determined to be necessary for cure.

This constellation of therapies was the foundation of the HLH-94 protocol, used in a large international pediatric trial. Etoposide, dexamethasone, and intrathecal methotrexate were used as an 8-week induction regimen, followed by intermittent etoposide, dexamethasone, and twice daily cyclosporine as a bridge to HCT (25). In a long-term follow up study, 29% of patients died before HCT, and for those that underwent HCT, the 5-year survival was 66%. There was a trend for improved HCT outcomes in patients with better disease control at the time of HCT. Early deaths were reported in patients who developed jaundice, edema, and renal dysfunction, suggestive of veno-occlusive disease (VOD). Even with the 20-30% mortality rate pre- and post-HCT, outcomes in this study were significantly improved from historic regimens. As a follow up, the HLH-2004 study started cyclosporine during induction, but this did not improve outcomes, and there were concerns regarding the side effects of cyclosporine (26).

In the modern era, etoposide and dexamethasone still serve as the foundation of pre-HCT induction therapy in primary HLH (27). Due to the risk of myelosuppression (and associated infection risk) as well as secondary malignancy (albeit rare) with etoposide, alternative agents have been explored. For example, emapalumab (IFN-γ antagonist) is now FDA approved for patients with primary HLH who have had a refractory, recurrent, or progressive course or intolerance to conventional therapy (28). Other options include alemtuzumab, a monoclonal antibody against CD52 (targets T cells, B cells, and macrophages) and ruxolitinib (janus kinase (JAK)1/2 inhibitor) (29). In practice, these agents are not mutually exclusive with etoposide and can be used sequentially or in conjunction. For example, as will be discussed below, one of the major barriers to HCT for HLH is the high rate of graft failure, particularly with reduced intensity conditioning (RIC) regimens. This may be due to a detrimental effect of IFN-γ on donor stem cells that cannot compete with residual recipient cells in the bone marrow that are ‘better acclimated’ to high-level IFN-γ environments (30). Bridging emapalumab in patients previously treated with etoposide may address this challenge and improve donor chimerism (30).

### Allogeneic HCT for primary HLH

Identifying the best conditioning regimen for HCT in HLH has been historically difficult. Initial transplants with busulfan (Bu)-based myeloablative conditioning (MAC) had high rates of fatal VOD, likely due to the hepatotoxicity of Bu in patients with pre-existing liver dysfunction from HLH. This motivated lower dose Bu or RIC regimens such as fludarabine/melphalan/alemtuzumab (Flu/Mel/Alem). While the toxicity of RIC regimens was lower, they had high rates of mixed chimerism and graft failure, with chimerism levels of at least 20-30% thought to be necessary to protect against HLH recurrence (31). A large study using the Center for International Blood and Marrow Transplant Research registry compared four different commonly used conditioning regimens, adjusted for year of transplant (32). These included Bu/Cyclophosphamide (Cy), Bu/Flu, Flu/Mel/Alem, and Flu/Mel/Alem/Thiotepa (TT). The primary outcome was event free survival (EFS) – i.e., survival without primary or secondary graft failure. While overall survival (OS) was similar between regimens, rates of graft failure were substantially higher in the Flu/Mel/Alem regimen, corresponding to the lowest EFS of 44% at 5 years. Addition of thiotepa (an alkylator) to this regimen substantially reduced graft failure, improved EFS, and was associated with a low rate of VOD, establishing Flu/Mel/Alem/TT as the currently recommended regimen.

As previously mentioned, CNS involvement occurs in a substantial fraction of patients with primary HLH, and in some forms of secondary HLH. Clinical symptoms may include seizures, altered mental status, and/or encephalopathy, with delayed or insufficient treatment risking permanent motor and cognitive deficits. Thus, early identification is crucial. As not all patients have clinical symptoms at onset, screening MRI and lumbar puncture are recommended (24). In addition to CNS inflammation being part of HLH, there is an entity of CNS-restricted HLH, often driven by similar pathogenic variants but more difficult to diagnose given the lack of systemic features and inflammatory markers (33). Compared to the classic systemic form of primary HLH, the onset of CNS-restricted HLH occurs later – at a median age of 6.5 years in one report (33). The differential diagnosis includes CNS vasculitis, acute disseminated encephalomyelitis, acute necrotizing encephalopathy, or CNS involvement of an underlying condition. Diffuse white matter and/or cerebellar involvement is commonly seen on imaging, and pleocytosis, elevated protein, and elevated neopterin may be seen in the cerebrospinal fluid (CSF). There are no consensus guidelines on treatment of CNS-restricted HLH but dexamethasone with or without intrathecal methotrexate may be considered, as can etoposide and emapalumab. Regardless of medical treatment choice, the definitive treatment of CNS-restricted HLH remains HCT, ideally as soon as feasible to avoid irreversible neurologic damage (34, 35).

### Summary and future directions

Therapeutic approaches for primary HLH, including first line and HCT regimens, are likely to continue to evolve, as will supportive care measures. Our group has recently considered pre-emptive (prior to HCT) tracheostomy for small patients considered to be at high risk for respiratory complications. While not part of any established guidelines, this has been beneficial for respiratory support through the acute transplant period. In parallel, *ex vivo* autologous hematopoietic stem cell-based gene therapy for genetically defined HLH is being developed. The first trial is opening in France for patients with *UNC13D* mutations (NCT06736080). While this approach has great potential, challenges include the harvesting of stem cells from critically ill patients, the requirement for therapy while the genetically manipulated product is manufactured, and uncertainty about which conditioning regimen will be required and tolerated to facilitate engraftment of the genetically manipulated product.

## HLH secondary to rheumatic disease

### Classification and pathophysiology

HLH secondary to rheumatic disease is referred to as macrophage activation syndrome (MAS), with an estimated 10% of cases of HLH/MAS attributable to this cause in children (14). By far, the rheumatic disease most associated with MAS in pediatrics is systemic juvenile idiopathic arthritis (sJIA), also known as Still’s disease. Systemic JIA presents uniquely from other subclasses of JIA with daily fevers, rash, lymphadenopathy, and potentially MAS [in up to 14% of cases (36)]. This systemic inflammation results from an autoinflammatory component to the disease pathophysiology, with disease-specific cytokine elevation, including IL-1 (37), IL-18 (38), and IL-6 (39). While arthritis is part of historic disease definitions (40), not all patients have arthritis at presentation, and with modern therapy, the development of arthritis may even be avoided (41). Patients are thus recognized as having sJIA based on other consistent disease features, including MAS, as well as sJIA-suggestive biomarkers described below.

The definition of MAS in sJIA is unique from primary HLH, with classification criteria shown in **Table 3** (42). The high incidence of MAS is hypothesized to result from elevations in IL-18 during active disease (43), which augments IFN-γ-production by cycling lymphocytes (6). These include HLADR+CD38^hi^ CD8+ T cells as well as CD4+ cells and NK cells, which have been found to be highly glycolytic due to activation of mTORC1 signaling. mTORC1 is a metabolic sensor that can drive cell proliferation and determine function. This mTORC1 signaling was also found in activated monocytes in murine models of sJIA and MAS, potentially coordinating the inflammatory response (44). In addition to IL-18 and IFN-γ signaling in sJIA-MAS, an elevated type I interferon signature has been described that may further prime pathogenic T and NK cells and augment IL-18 production (6). As type I interferons are commonly induced by viral infection, this may explain infection as a trigger of sJIA-MAS. Lastly, it has also been observed that heterozygous variants in primary HLH related genes are enriched in patients with sJIA who develop MAS (45) (**Figure 1**).

**Table 3:** Classification of MAS in sJIA (2016 criteria) (42).

A febrile patient with known or suspected sJIA is classified as having MAS if the following criteria are met:
Ferritin > 684 ng/mL **AND any 2 of the following:**
Platelet count ≤181 × 10^9^/liter
Aspartate aminotransferase >48 units/liter
Triglycerides > 156 mg/dl
Fibrinogen ≤ 360 mg/dl

*73% sensitivity and 99% specificity.

### Medical treatment of sJIA and sJIA-MAS

The American College of Rheumatology recommendation (46) for first line treatment of sJIA is IL-1 inhibition (anakinra, canakinumab) or IL-6 inhibition (tocilizumab) (47–50). Use of JAK inhibitors in more severe cases, is increasingly common (51, 52). As plasma IL-1 levels are not reflective of IL-1 secretion, due to greater production in the tissues and binding to a natural decoy receptor in the blood (53), IL-18 – a related cytokine – is instead monitored. In active sJIA, IL-18 levels usually range from 10^4^ to 10^5^ pg/mL, which can help identify sJIA from other febrile disorders (38). During episodes of MAS, IL-18 levels >10^5^ pg/mL are often observed, and IFN-γ related biomarkers (e.g. CXCL9) become significantly elevated (39).

Unlike in primary HLH, there are few dedicated trials or consensus guidelines for treatment of MAS (14). To create a framework for approaching MAS/HLH diagnosis and treatment, our center previously developed an Evidence Based Guideline (EBG) based on expert participation from diverse specialties at Boston Children’s Hospital, using a consensus-based process (54, 55). This led to treatment recommendations that include consideration of anakinra, intravenous immune globulin, calcineurin inhibitors, and high dose steroids for MAS. Since the implementation of this EBG, there has been updated evidence for use of JAK inhibitors (for other etiologies of HLH) (29) and emapalumab (recently FDA approved for MAS) (56), and the EBG has been updated accordingly. Use of etoposide, although described and beneficial for some patients with sJIA-MAS, is generally not recommended due to associated myelosuppression but could be considered in collaboration with Oncologists for patients with highly refractory disease (57, 58). A 2025 review on treatment evidence for MAS is provided (57). Importantly, while systemic reviews and single center guidelines are important reference tools, centers should adapt recommendations to local resources and patient specific needs. For example, general dosing guidelines may not be appropriate for patients with significant organ dysfunction or in the setting of potential drug-drug interactions (e.g. liver toxicity and/or antifungal prophylaxis with ‘azoles’ for ruxolitinib). Infection prophylaxis should be considered but is not detailed in this review. Lastly, with new advances in the field, guidelines will eventually become outdated.

### Autologous and allogeneic HCT for sJIA and sJIA-MAS

Beyond treating individual episodes of MAS, there is also a history of HCT for sJIA, although transplant strategies and indications have evolved over time. Initially, in the late 1990s when there were no sJIA targeted biologics, autologous HCT was explored for patients with refractory arthritis. The premise was that high dose chemotherapy (usually ATG and Cy ± Flu or low-dose total body irradiation) could reset the immune system, and pre-collected hematopoietic stem cells would then be infused to restore hematopoiesis with subsequent production of naïve, clonally diverse T cells in the thymus. Intrinsic to this approach was *ex vivo* T cell depletion of the stem cell graft to avoid reinfusing dysregulated T cells. In a multicenter cohort of 34 patients with arthritis unresponsive to methotrexate and at least one other therapy, 53% of patients had a complete response, 18% a partial response, and 21% had no response by an average of 29 months of follow up (59). Prolonged depression of CD4+ T cells was common after transplant. Three patients developed fatal MAS triggered by infection, prompting an international consensus to reduce the degree of T cell depletion and improve disease control ahead of transplantation (60). Given the transplant related mortality of 9% and the emergence of IL-1 and IL-6 targeted biologics by the mid-2000s, autologous HCT became uncommon.

**Figure 2:**
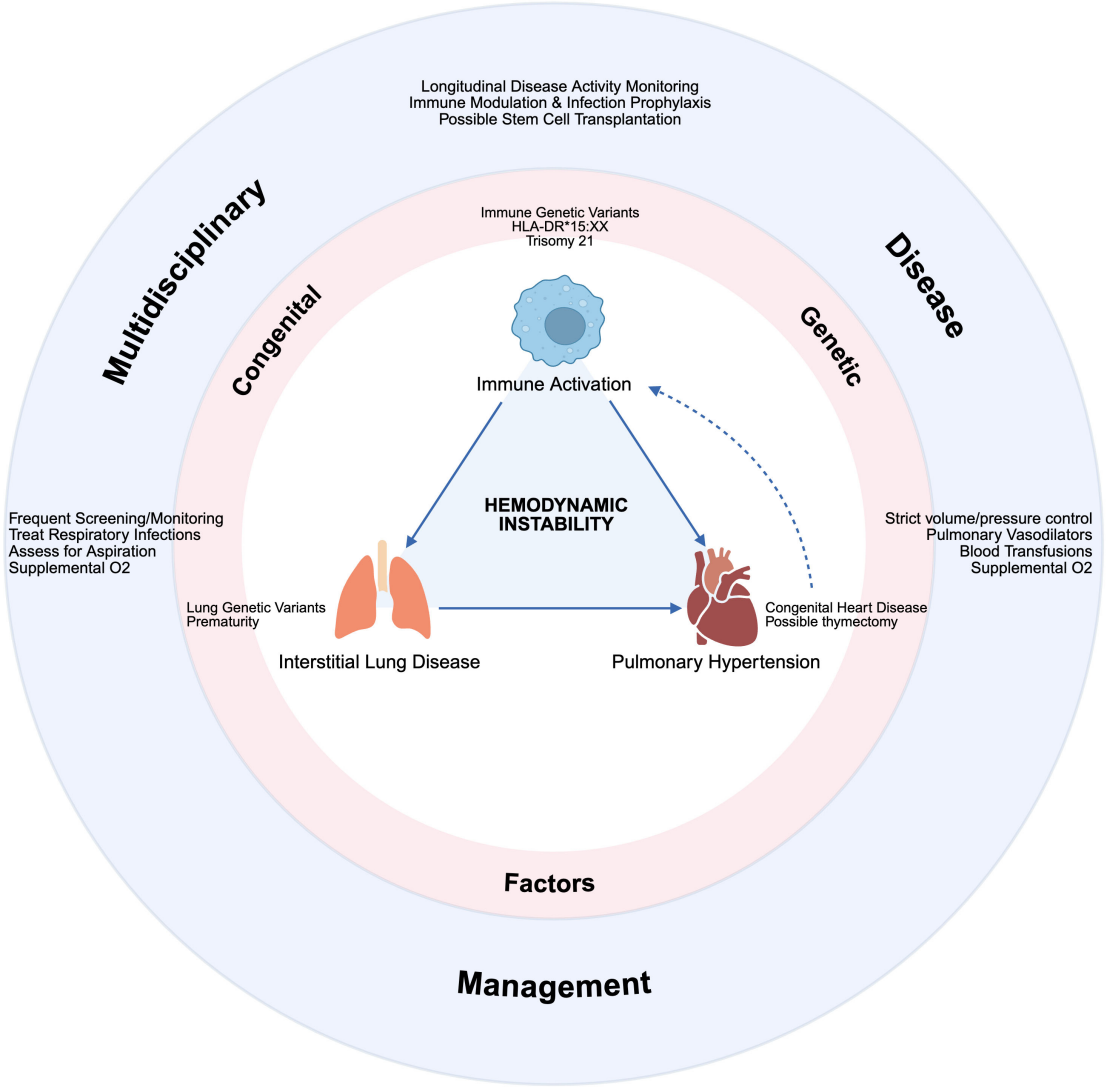
Schematic of the multi-organ involvement in sJIA and MAS, often requiring critical care management in collaboration with rheumatology, pulmonology, cardiology, and stem cell transplantation. Created in BioRender.

With new biologic agents curtailing the development of chronic arthritis, morbidity from sJIA transitioned from arthritis to recurrent MAS and potentially fatal lung disease (sJIA-LD), comprised of interstitial involvement and/or pulmonary hypertension (61). One of the earliest reports of sJIA-LD had a 5-year survival of only 42% (61). Due to increased screening, patients with less severe lung disease are now being identified, improving survival statistics (62). Risk factors for lung disease include young age of sJIA onset, HLA-DRB1*15 positivity, congenital heart disease, chronic IL-18 signaling, recurrent MAS, and trisomy 21 (**Figure 2**) (63–66). The latter two factors have a causal explanation – an IFN-γ signature has been found in the bronchoalveolar lavage fluid of patients with lung disease suggesting a role in its pathogenesis (67). MAS is largely IFN-γ driven, and in trisomy 21, there is increased interferon signaling due to triplicate copies of a receptor for IFN-γ (IFNGR2) (68). Due to concern for disease relapse and MAS flare with autologous HCT, the field shifted to allogeneic HCT for recurrent MAS ± lung disease as the main transplant indications. There have been two retrospective cohort studies in the last 10 years. The first included 11 patients with treatment-refractory sJIA with 5/11 having a history of MAS (69). All patients received a reduced toxicity conditioning (RTC) regimen of Flu/Mel/Alem or Flu/Treosulfan/Alem. Viral infections after transplant were common and 1/11 patients died of fungemia in the setting of graft-vs-host-disease treatment. At last follow-up, 9/10 surviving patients were in complete remission (no symptoms, off immunosuppression), while one patient had a flare of arthritis and an MAS-like episode. The second report included 13 patients with sJIA-LD – 5 with an oxygen requirement prior to transplant (70). Patients received RIC or RTC regimens, most commonly Flu/Mel/Alem/TT (akin to that used in primary HLH). Four patients died – two from cytomegalovirus infection, one from intracranial hemorrhage during asymptomatic SAR-CoV2 infection, and one from progressive sJIA-LD. All 9 surviving patients were in complete remission off immune suppression at the time of last follow up, with previously oxygen-dependent patients coming off oxygen (70).

### Summary and future directions

In summary, the pediatric rheumatic disease most associated with MAS is sJIA due to baseline activation of pathways that can trigger MAS-related inflammation. Medical management with small molecules and biologic agents may successfully control these episodes in some patients, but for those with recurrent MAS and/or lung disease, allogeneic HCT may be considered to halt a potentially fatal disease trajectory and achieve drug free remission. As transplant-related mortality remains 12.5-25% (higher for those with lung disease), careful patient selection is required, and there is currently no consensus on when this should be pursued.

## HLH secondary to infection

### Classification and pathophysiology

Infections are the most common trigger of HLH in children, both in primary and secondary cases (57% of cases overall) (14). Viral, bacterial, parasitic, and fungal infections have been linked to HLH in both immune competent and immune compromised hosts. Work-up for suspected HLH should therefore include comprehensive evaluation in consultation with an infectious disease expert. As HLH is an uncommon complication of infection overall, a genetic evaluation should be strongly considered to look for predisposing pathogenic variants.

The most common trigger of infection associated HLH is EBV. EBV is a herpesvirus that primarily infects B cells through their CD21 and CD35 receptors and establishes latency. Initial EBV infection is asymptomatic in most individuals, and an estimated >90% of adults are latently infected (71). Clinical sequelae can range from ‘infectious mononucleosis’ (e.g., fever, sore throat, splenomegaly) to EBV-related malignancy or HLH.

A recent study by Liu et al. elucidates why EBV has a higher predilection for triggering HLH (72). In this study, serum and peripheral blood mononuclear cells were compared amongst pediatric patients with non-EBV viral illness, EBV infection, and known HLH. Relative to other viral illnesses, acute EBV infection led to increases in IL-18 and IL-27 (which contribute to Th1 cell polarization) as well as CXCL9. As with other etiologies of HLH, there was also expansion of HLADR+CD38^hi^ CD8+ T cells, which corresponded to the ‘atypical lymphocytes’ often observed in acute EBV infection (72). Overall, these data suggest that the response to acute EBV infection includes production of cytokines and populations of T cells that are hallmarks of HLH.

### Medical treatment of infection-driven HLH

Treatment of infection-driven HLH involves targeting the underlying infection in addition to standard HLH directed therapies. For EBV driven HLH, addition of a B cell targeting agent like rituximab has been shown to facilitate elimination of the EBV reservoir (73). Anakinra is safe in the setting of infection (was initially investigated for patients with sepsis) (74, 75) but is not always sufficient to control EBV-HLH (76). In a single-arm phase II trial, 52 pediatric patients with a new diagnosis of HLH (diverse etiologies but malignancy excluded) received ruxolitinib as first-line therapy, with a complete response of 58.3% in the subset with EBV-HLH (29). Lastly, emapalumab may be used as an adjunctive agent to chemotherapy for cases that are particularly severe (77, 78). More specific guidelines for EBV-HLH will require prospective studies. However, one important consideration is whether a patient is at risk for recurrence.

### Allogeneic HCT for CAEBV and EBV-predisposing IEIs

Allogeneic HCT may be needed to resolve EBV viremia and its associated complications. For example, there are several IEIs (due to loss-of-function variants in XIAP, SAP, CD27, CD70, ITK, and MAGT1) associated with EBV-HLH and EBV-triggered lymphoma (79) that impair the ability of the immune system to clear EBV infection. There is also an entity known as Chronic Active EBV (CAEBV), which describes persistent EBV viremia without a known IEI. CAEBV has clear geographic associations and has mainly been described in East Asia and in Central America. As with some IEIs, a characteristic of CAEBV is that EBV infection is found in other lymphoid and myeloid lineages – thus not restricted to the B cell compartment (**Table 4**). A 2024 study by *Wang* et al. provides a potential explanation (80). In this study, bone marrow and peripheral blood samples were obtained from patients with CAEBV, supporting infection of hematopoietic stem cells, which then differentiate to become EBV infected myeloid and lymphoid cells. While the circumstances under which they become infected with EBV are still unknown, this finding supports allogeneic HCT as the only curative option. There is no standardized treatment regimen for CAEBV, but a combination of immune targeted therapy (e.g. steroids, cyclosporine, PD1 inhibition) and/or chemotherapy is often used to control disease ahead of HCT (81, 82). In a large Japanese registry study of 102 patients with CAEBV that underwent allogeneic HCT, worse outcomes were associated with elevated soluble IL2 receptor at the time of transplant (suggesting poor control of inflammation) and use of radiotherapy in conditioning regimens (83). In a smaller study, MAC and RIC regimens were compared (84). The EFS at 3 years was 54.5 ± 15.0% in the MAC group and 85.0 ± 8.0% for the RIC group, supporting use of the latter.

**Table 4:** Diagnostic criteria for chronic active EBV (81).

A patient may be diagnosed with CAEBV if all 4 criteria are met
1. Persistent or recurrent infectious mononucleosis-like symptoms for more than 3 months 2. Detection of an increased number of EBV genomes in peripheral blood and/or affected tissues 3. Detection of EBV-infected T or NK cells in peripheral blood and/or affected tissues 4. Chronic illness that cannot be explained by other known disease processes at the time of diagnosis

## HLH associated with malignancy

### Classification and pathophysiology

HLH may be seen secondary to hematologic malignancies, which is a substantial challenge and major driver of HLH in adult patients (14) but observed in children more rarely (85). HLH may occur at diagnosis and be driven by the malignancy (M-HLH) or during chemotherapy (Ch-HLH). Ch-HLH or “on-therapy” HLH should raise high concern for a concomitant infection. Limited data on the frequency and management of malignancy associated HLH in pediatrics is available, although malignancy is estimated as the underlying etiology in approximately 5% of HLH cases (14). In one retrospective analysis of 29 pediatric and young adult patients, HLH was considered triggered by the malignancy in 21 patients with T- (n=12) or B-cell neoplasms (n=7), with EBV infection present as a co-trigger in 5 patients. The remaining 8 patients experienced HLH during chemotherapy (Ch-HLH) mainly for acute leukemias (86).

The diagnosis of M-HLH is frequently difficult to make, particularly when the underlying malignancy is yet undiagnosed. The diagnosis of M-HLH should be considered in a patient with a biopsy-proven malignancy who has clinical, and laboratory features consistent with HLH and no alternative cause of hyperinflammation. Different criteria and scoring systems have been used in adult patients, including the MD Anderson Criteria for M-HLH, H Score, and modification to the HLH-2004 criteria (87). Additionally, an Optimized HLH Inflammatory index (OHI) comprising soluble CD25 >3900 U/mL and Ferritin >1000ng/mL was developed to diagnose M-HLH more accurately and predict mortality in adult patients (88).

### Medical treatment of malignancy associated HLH

Current treatment practices for M-HLH are largely based on expert opinion, given the absence of randomized clinical trials and heterogeneity of patient populations. Treatment is centered on the initiation of malignancy-directed therapy as soon as safely feasible and utilizing anti-inflammatory therapies to control hyperinflammation. In a pediatric cohort, the median overall survival (OS) was 1.2 years in M-HLH and 0.9 years in Ch-HLH (86). Given this was a small retrospective series, the superiority of prioritizing malignancy-directed vs. HLH-directed therapies could not be determined for the M-HLH cohort. In the Ch-HLH cohort, interventions ranged from postponement of chemotherapy to the use of etoposide-containing regimens (86). Adult M-HLH may be managed with an etoposide-based regimen that closely resembles the HLH-94 protocol, consisting of 8 weeks of treatment with dexamethasone 10mg/m^2^/d, subsequently tapered, or a dose-reduced regimen in patients with advanced age or significant comorbidities (89). A single-arm Phase 2 trial evaluated the safety and efficacy of a dose adjusted EPOCH regimen (Etoposide, Prednisolone, Vincristine, Cyclophosphamide, Doxorubicin) with or without rituximab in patients with previously untreated Non-Hodgkin Lymphoma-associated HLH to simultaneously address the HLH and underlying lymphoma. While this regimen led to an overall response rate (ORR) of 80.7% and 5-year OS of 73.1% in patients with B-cell lymphomas, those with T/NK lymphomas had dismal outcomes, with ORR of 13.8% and 1 year OS of 3.4% (90). A two-tiered approach of using etoposide and corticosteroids and, if needed, a salvage regimen of doxorubicin, etoposide, and methylprednisolone, to control the HLH with subsequent initiation of appropriate chemotherapy is frequently favored. Allogeneic HCT or cellular therapy in patients with malignancy associated HLH are employed only if this is indicated for the underlying malignancy.

## HLH associated with IEC therapy

In contrast to HLH-syndromes in which an underlying predisposition and trigger need to be rapidly diagnosed and controlled, novel immunotherapeutic strategies purposefully trigger and employ the inflammatory capacities of the immune system to eradicate malignancies (91), and more recently, to address autoimmune conditions (92–94). IEC products can be generated by genetic modification of autologous or allogeneic T or NK cells to express Chimeric Antigen Receptors (CARs) that target cell-surface antigens. Similarly, genetically modified T cell receptors target intracellular antigens (TCR-T cells). With a growing number of FDA approved CAR T cell products and hundreds of active clinical trials for an expanding number of disease indications, we will focus on hyperinflammatory sequelae of CAR T cells below.

### CRS

The earliest recognized hyperinflammatory complication of CAR T cell therapy was Cytokine Release Syndrome (CRS) (95). The hallmarks of CRS are fever, cardiopulmonary dysfunction, and other (hepatic, renal, gastrointestinal) organ dysfunction. Laboratory abnormalities include elevations in ferritin and IL-6, and coagulopathy, resembling those of HLH/MAS. CAR T cell activation leads to proliferation, secretion of proinflammatory cytokines, and activation of the host immune system including myeloid cells, thereby inducing an inflammatory feedback loop. In patients receiving 4-1BB costimulated CD19-CAR T cells, elevations in IFN-γ, IL-6, IL-8, sIL-2R, sgp130, MCP1, MIP1α, MIP1β, and GM-CSF as well as higher ferritin (>10,000) and CRP were significantly associated with severe (Grade 4-5) CRS compared to those with mild (Grade 0-3) CRS (96). Risk factors for the severity of CRS include cell dose, as well as recipient characteristics such as high tumor burden (97). Additionally, pre-infusion inflammation, peak expansion kinetics, and characteristics of the CAR itself are important (98) such as the transmembrane domain, costimulatory domain (e.g. CD28>41BB for CRS) (99) and affinity of the binder to target (less CRS with lower affinity binder) (100). Thus far, patients receiving CAR T cells for autoimmune indications have not been shown to be at increased risk for CRS, and rates and severity of CRS appear to be low (101). However, patients can experience localized inflammation in previously affected tissues, which is often mild and self resolves or resolves with steroid therapy (referred to as localized immune effector cell associated-toxicity syndrome or LICATS) (102).

The use of the monoclonal antibody tocilizumab revolutionized CRS management by interrupting the proinflammatory signaling cascade via IL-6 blockade without impacting CAR T cell efficacy (95, 103). While corticosteroids were initially avoided so as not to impair function and durability of CAR T cells, targeted use of corticosteroids during peak inflammation or prophylactically in high-risk patients has not been found to adversely impact efficacy (104).

### ICANS

A related hyperinflammatory syndrome, IEC-associated neurotoxicity syndrome (ICANS), was recognized as a distinct entity that could overlap with CRS or occur following its resolution, and thus distinct grading systems and management algorithms were developed (105). Symptoms may range from mild confusion, headaches, tremors, and word finding difficulties to focal deficits, seizures, coma, and cerebral edema. Particularly in patients with prior CNS involvement or pre-existing neurological deficits, documentation of a patient’s baseline neurological exam and, if indicated, baseline MRI/imaging prior to CAR T administration is critical. Management guidelines suggest expeditious workup with neuroimaging, lumbar puncture, and EEG, in consultation with a Neurology team. Preclinical studies have identified IL-1 and IL-6 as key mediators in CRS and ICANS. However, while IL-6 blockade ameliorates CRS, only IL-1 blockade abrogates both CRS and ICANS (106–108). Initially treated supportively, management of ICANS currently includes corticosteroids, tocilizumab (if co-occurring with CRS, although there are concerns about enhancing IL-6 levels in the CSF due to non-CSF permeability of tocilizumab) and, if refractory, anakinra, intrathecal chemotherapy, and CSF pressure relief (109–111).

### IEC-HS

Most recently, a separate entity, IEC-associated hemophagocytic lymphohistiocytosis-like syndrome (IEC-HS), was described following the experience with CD22-CAR T cell therapies (112). This syndrome has substantial overlap with the clinical and laboratory features of CRS and may be difficult to distinguish; however, it typically presents with delayed onset or following resolution of initial CRS and is not responsive to conventional CRS management with tocilizumab. Although the frequency was as high as 40.4% in a trial of 59 patients infused with CD22-CAR T cell products (112), other CD22-CAR T cell trials reported lower rates at 18.75% (113). A broad consensus definition (**Table 5**), grading system, and management recommendations have since been developed by the American Society for Transplantation and Cellular Therapy (114). With increasing recognition, it has also been described with other CAR T cell products such as those targeting CD19 (115) and in investigational CAR T cell products for solid tumors (116). Onset with resolving/resolved CRS or a worsening inflammatory response after initial improvement with CRS therapy is one of the most common manifestations of IEC-HS. Pre-infusion NK cell lymphopenia and higher bone marrow T cell:NK cell ratio may predispose to IEC-HS at least in the setting of CD22-CAR T cell therapy, possibly leading to a deficiency in regulating CD8+ T cell hyperactivation and CAR T cell contraction (112). Free IL-18 was differentially elevated in patients with CRS and IEC-HS compared to those with CRS alone (117) and may warrant investigation of agents targeting this pathway such as tadekinig alfa, a human recombinant IL-18-binding protein, which is not currently FDA approved. High pre-CAR T cell disease burden, a high level of pre-infusion inflammation based on ferritin and C-reactive protein, as well as lower platelet and neutrophil counts were associated with the development of IEC-HS in the context of the commercial CD19-CAR T cell product tisagenlecleucel (115). Germline genetic variants associated with HLH (118) and clonal hematopoiesis (119) may also contribute. Murine studies with perforin-deficient CAR T cells aimed at providing mechanistic insight, recapitulated elevated IL-1β and IL-18 levels and biphasic late CAR T cell expansion, which is characteristic of IEC-HS in humans (120). However, whether the pathophysiology underlying IEC-HS is distinct from or on the spectrum of CRS, and the identification of unifying risk factors, remains an area of active investigation. Similarly, the optimal therapeutic approach remains to be defined. Anakinra is recommended as first-line therapy given its favorable side-effect profile and short half-life allowing dose titration. Corticosteroids should be incorporated for moderate or higher disease severity or maintained in patients already receiving them for CRS. Given the importance of the IFN-γ signaling pathway in IEC-HS, as in other HLH-like conditions, ruxolitinib or emapalumab (used more frequently in pediatrics) are being employed for high grade/progressive IEC-HS (114, 121). Although the effect on durable CAR T cell function remains to be clarified in clinical studies, preclinical models predict that in hematologic malignancies, IFN-γ blockade can reduce macrophage activation without compromising CAR T cell function (122, 123). In contrast, IFN-γR signaling was shown to play a critical role in the susceptibility of solid tumors such as glioblastoma to CAR T cell mediated killing (124). This highlights the need for judicious use of these agents and further investigation to elucidate their impact mechanistically. Given the high rate of morbidity and mortality associated with IEC-HS, prompt diagnosis and aggressive management should be instituted.

**Table 5:** Diagnostic criteria for IEC-associated HLH-like syndrome (114).

**Definition of IEC-HS**	The development of a pathological and biochemical hyperinflammatory syndrome independent from CRS and ICANS that 1) manifests with features of macrophage activation/HLH, 2) is attributable to IEC therapy, and 3) is associated with progression or new onset of cytopenias, hyperferritinemia, coagulopathy, and/or transaminitis
Criteria for identifying IEC-HS^*^	Clinical/Laboratory Manifestations
Most common manifestations** ULN = Upper limit of normal; LLN = Lower limit of normal	Required: Elevated ferritin (>2 x ULN or baseline (at time of infusion)) and/or rapidly rising (per clinical assessment)
	Onset with resolving/resolved CRS or worsening inflammatory response after initial improvement with CRS-directed therapy***
	Hepatic transaminase elevation^Δ^ (> 5x ULN (if baseline was normal) or >5x baseline (if baseline was abnormal)
	Hypofibrinogenemia (<150mg/dL or <LLN) ^ΔΔ^
	Hemophagocytosis in bone marrow or other tissue^ΔΔ^
	Cytopenias (new onset, worsening or refractory) ^ΔΔΔ^
Other manifestations that may be present	Lactate dehydrogenase elevations (>ULN)
	Other coagulation abnormalities (e.g. elevated PT/PTT)
	Direct hyperbilirubinemia
	New-onset splenomegaly
	Fever (new^#^ or persistent) ^ΔΔ^
	Neurotoxicity
	Pulmonary manifestations (e.g. hypoxia, pulmonary infiltrates, pulmonary edema)
	Renal insufficiency (new onset)
	Hypertriglyceridemia (fasting level, >265 mg/dL) ^ΔΔ^

*Diagnosis was made only when not attributable to alternative etiologies, including CRS, infection and/or disease progression. **Constellation of findings typically simultaneously (e.g., all within 72hrs). ***Although most cases of IEC-HS have been seen with antecedent CRS, this may not always be the case, and emerging experience will shed light on how IEC-HS may present. ^Δ^Consistent with grade 3 hepatic transaminase elevations according to Common Terminology for Adverse Events version 5.0. ^ΔΔ^According to HLH-2004. ^ΔΔΔ^Generally, at least 1 lineage will be a grade 4 cytopenia (platelets, neutrophils, hemoglobin). ^#^As distinguished from CRS onset or recrudescence.

## Diagnostic considerations in the critical care setting

Although dramatic advances have been made in the recognition, diagnostic workup, and treatment of HLH since the recognition of primary HLH in the 1950s and secondary HLH in the late 1970s, this life-threatening disorder remains underdiagnosed (125). A high degree of suspicion is required to expeditiously initiate diagnostic pathways for primary and secondary HLH syndromes and embark on appropriate treatment strategies, with consideration of the underlying etiology. All critically ill patients should have complete blood count testing, and multilineage cytopenias in a febrile patient should increase suspicion for HLH and prompt a ferritin to be sent if not already. Transaminitis, coagulopathy, and hepatosplenomegaly may also increase suspicion. If the ferritin is significantly elevated, this may trigger more specific markers of HLH to be sent such as CXCL9. The clinical context of fever and hyperinflammation is also important, as a patient with a history of sJIA, for example, is more likely to have sJIA-MAS, and a patient with symptoms of EBV may prompt consideration of EBV-HLH (vs. sepsis). Meanwhile, patients with positive blood cultures or suspicious sources for bacterial inoculation increase the relative suspicion for sepsis.

If initial workup raises suspicion for primary or secondary HLH, further diagnostic testing should be undertaken in consultation with rheumatologists, oncologists, immunologists, infectious disease specialists, neurologists (if any neurologic symptoms or deficits are present), and stem cell transplant/cell therapy physicians. Workup should extend beyond classic familial HLH or EBV susceptibility disorders, given that HLH may present as the first manifestation of a diverse spectrum of IEI, often with an unrecognized history of infections (126). Diagnostic workup and timely management should be rapidly instituted to prevent morbidity and mortality, even if diagnostic certainty is not yet achieved or the underlying genetic defect remains under investigation. Considerations for the multidisciplinary workup of HLH and HLH-like syndromes are summarized in **Table 6**.

**Table 6:** Diagnostic considerations for suspected HLH-disorders.

Diagnostic Workup considerations for suspected HLH-disorders				
Initial Workup for Unknown Etiology				IEC therapy
**Laboratory Studies:** CBC w/diff, Bili/LFTs, Ferritin, soluble IL-2R (sCD25), Coagulation studies including Fibrinogen, Triglycerides. Consider: CXCL-9, IL-18, CRP, ESR, LDH, Procalcitonin, Cytokine Panel if available **Infectious Workup:** Blood cultures, EBV/CMV/Adenovirus PCRs, additional testing based on patient presentation and exposure history **Bone Marrow Aspirate/Biopsy:** Assess for hemophagocytosis (may defer if secondary HLH suspected; supports but is not essential for diagnosis of HLH) **Imaging:** CT of Neck, Chest, Abdomen, Pelvis or CXR and Abdominal US as indicated **CNS assessment:** particularly for primary HLH if safe (defer with coagulopathy)**;** MRI brain (for parenchymal lesions, diffuse edema, leptomeningeal enhancement, periventricular white matter changes, volume loss or spine lesions; note, normal MRI does not rule out CNS-involvement), lumbar puncture with CSF analysis (cell count, protein, glucose, pathology interpretation, neopterin, infectious studies if indicated)				Baseline Assessments: - Disease burden - Cardiac function - Renal function - Neurologic exam +/-MRI - CBC w/diff - Chem 10 - LFTs/Bili - CRP/Ferritin - LDH - Cytokine panel (if available)

Further workup for suspected HLH				Monitoring
Primary HLH	Secondary HLH			
Genetic	Rheumatic	Infection	Malignancy	IEC
**Assess for genetic underpinnings of primary HLH in consultation with Oncology:** - Perforin (by FC*) - SAP (if male, by FC) - XIAP (male and some female, by FC) - CD107a-degranulation (FC)/NK Cytotoxicity - Hair analysis (pigment abnormalities) - Targeted genetic panel or WES **Evaluation for IEI in consultation with Immunology/Genetics:** - Lymphocyte subsets - T cell phenotyping - IgG, IgA, IgM titers - Vaccine titers - Neutrophil Oxidative Burst (DHR) - Targeted genetic panel or WES - Metabolic workup if indicated **Preparation for HCT:** - HLA typing and initiation of donor search	**Assess for underlying autoimmune or autoinflammatory condition in consultation with Rheumatology:** - Disease specific evaluation by history/exam (e.g. for sJIA, SLE, JDM) - IL-18, CXCL9 - May pursue genetic testing if history of immune dysregulation suggestive of an IEI -Cycling lymphocytes (CD38+/HLA-DR+ by FC)	**Assess for underlying infection in consultation with ID:** - Viral: HSV, VZV, HHV6/8, HIV, parvovirus, others as indicated - SARS-CoV-2 - Leishmania PCR if indicated - Tick or Mosquito-Borne Disease Testing - TB, Histoplasmosis, others if indicated **Evaluation for IEI in consultation with Immunology/Genetics (see primary HLH/Genetic)**	**Assess for underlying malignancy in consultation with Oncology:** - Imaging including PET-CT to evaluate for lymphoma - Biopsy of any suspicious imaging findings - Bone marrow aspirate/biopsy if not performed already - Determine OHI index (sCD25/Ferritin) to aid in diagnosis and prognosticate - Determine % activated CD8+CD38+HLA-DR+ lymphocytes to distinguish HLH from sepsis Consideration in patients undergoing chemotherapy	**Regular monitoring:** - CBC w/diff - Chem 10 - LFTs/Bili - CRP/Ferritin - Cytokine panel (if available) - Coags w/Fibrinogen - LDH - Vital signs - Signs of organ dysfunction **Concern for IEC-HS:** - Triglycerides - IL18 if available - CXCL9 if available - Increase monitoring frequency

*FC, flow cytometry; WES, whole exome sequencing; TB, tuberculosis.

## Management considerations in the critical care setting

**Figure 3:**
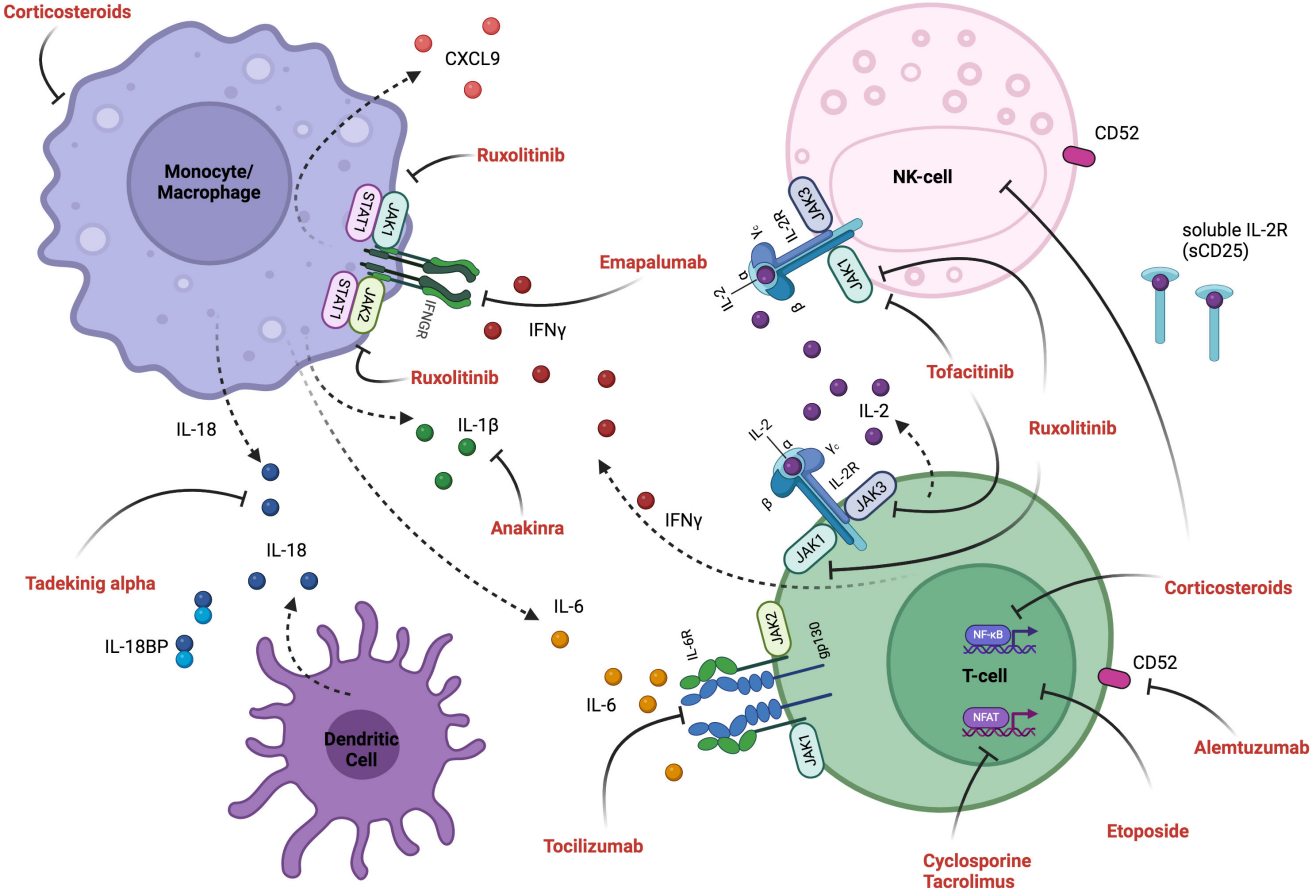
Inflammatory pathways and mechanism of therapeutic interventions. Activated T-cells produce pro-inflammatory cytokines (including IL-2, IFN-γ, TNF-α and GM-CSF), and their interaction with activated myeloid cells (producing IL-6, IL-18, IL-1β and CXCL9) induces an inflammatory feedback loop, which can be targeted therapeutically. See **Figure 4** for detailed explanation of targeted therapeutics and **Table 7** for cytokine descriptions. Created in BioRender.

**Figure 4:**
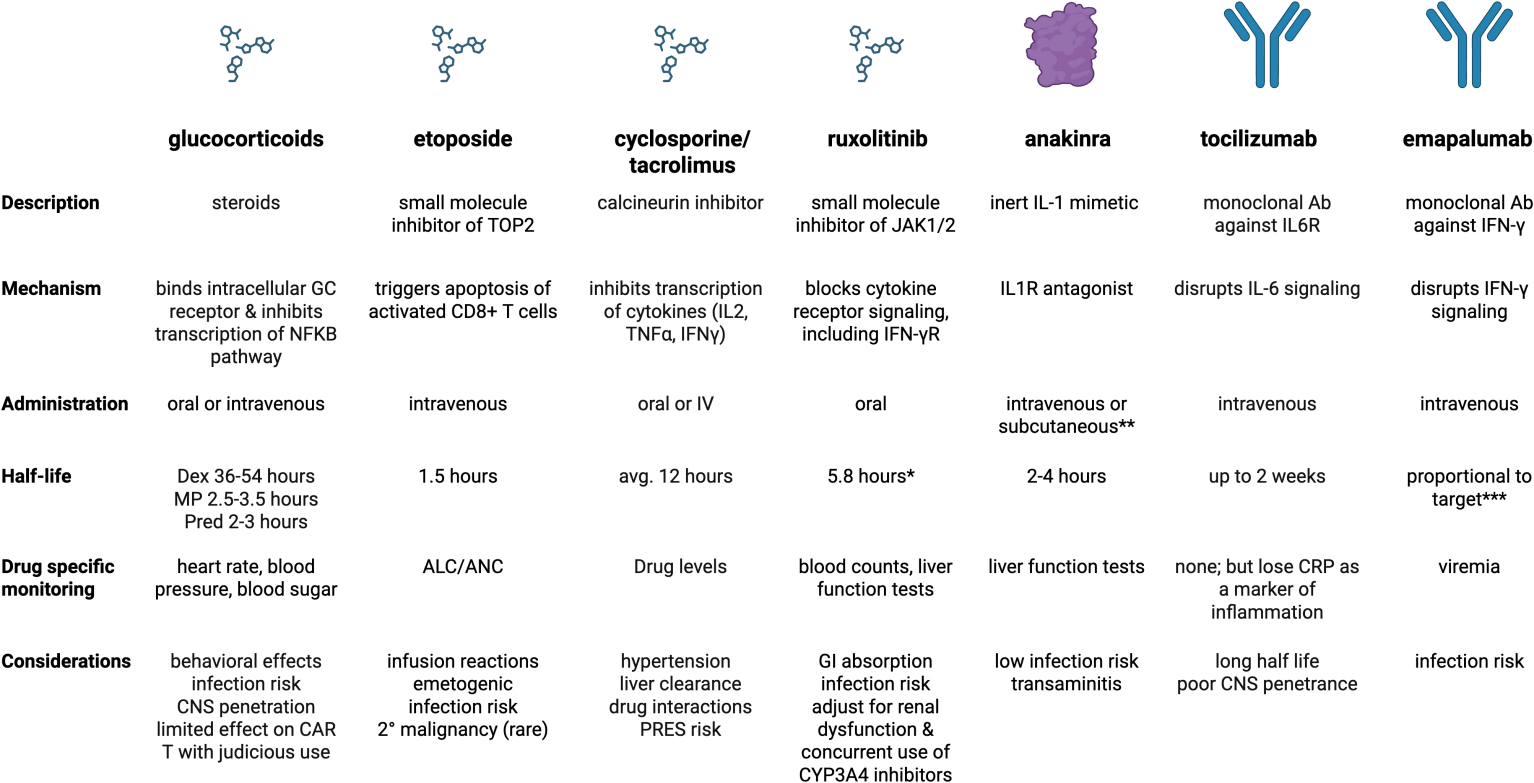
Summary of medications core to management of primary and secondary HLH. *includes ruxolitinib and active metabolites **subcutaneous form used more for outpatient management of sJIA ***clearance is proportional to total serum IFN-γ concentration when it is > 10,000 pg/mL (constant elimination if less). Created in BioRender. Dex, dexamethasone; MP, methylprednisolone; Pred, prednisone

The overarching principles of treatment include gaining control of the hyperinflammatory feedback loop with immunosuppressive, cytokine-modulating, and chemotherapeutic agents (**Figure 3**, **Table 7**, **Figure 4**), providing supportive care, and treating the primary etiology as well as modifying factors as soon as safely feasible (127). **Table 8** summarizes management considerations based on underlying etiology. In patients with EBV-driven HLH, EBV-directed therapy including rituximab to target CD20+ reservoirs of EBV should be employed. In patients with primary HLH, HLA-typing and donor workup should be undertaken to proceed to an allogeneic HCT once HLH is optimally controlled and HLH-directed therapy has been tapered to continuation therapy. Allogeneic HCT is also frequently indicated as the definitive therapy for IEIs presenting with HLH (often manifesting with an infectious trigger), CAEBV, and patients with recurrent MAS from sJIA, with similar goals of having controlled infection/inflammation at the time of transplant and potentially the use of reduced toxicity conditioning regimens to limit transplant related morbidity and mortality.

**Table 7:** Cytokine signaling in HLH.

Cytokine	Source	Receptor	Signaling	Effect
IL-2 (133, 134)	Primarily activated CD4+ T cells and, to a lesser extent, CD8+ T cells, NKT cells, activated dendritic cells, and mast cells	Binds to IL-2R consisting of IL-2Rα (CD25), IL-2Rβ (CD122), and the common γ chain (CD132). Soluble IL-2Rα is secreted by T cells and NK cells upon activation.	Activation leads to JAK (1/3)-STAT(5a, 5b). Also leads to PI3K/Akt and MAPK signaling.	Autocrine effect on T cell proliferation, survival and differentiation and NK-cell activation.
IFN-γ (8, 135)	Activated T cells (esp. CD8+ T cells) and NK cells with some secretion by APCs. Production is stimulated by IL-12, IL-15, and IL-18, and negatively regulated by glucocorticoids, IL-4, IL-10 and TGF-β	Upon binding of IFN-γ, IFNGR1 (ligand-binding subunit) and IFNGR2 (required for signaling) dimerize to form a heterodimeric receptor	Activation of JAK(1/2)-STAT (1) signaling	Activation and proliferation of CD8+ T cells. MHC-II upregulation on APCs, and activation of macrophages. IFN-γ polarizes macrophages towards an M1 phenotype characterized by enhanced phagocytosis, antigen presentation and production of pro-inflammatory cytokines including TNF, IL-1β, IL-6, IL-12, CXCL9 and IL-18.
IL-6 (95, 136)	Macrophages (main producers) in the context of IEC-associated hyperinflammation, although other immune cells, endothelial cells, and fibroblasts can produce	The IL-6 receptor consists of an IL-6 receptor subunit (IL6-R) and IL-6 signaling transducer gp130. When IL-6 levels are low, ‘classic’ signaling occurs when IL-6 binds to IL-6R expressing cells (macrophages, neutrophils, T-cells and hepatocytes), When IL-6 levels are elevated, ‘trans’ signaling can occur, in which IL-6 binds to soluble IL6R, which can associate with gp130 on a broad array of cells	JAK(1/2)/TYK-STAT (3) signaling.	Pleiotropic activity in many tissues. It induces synthesis of acute phase proteins such as CRP, inhibits the production of Albumin, induces the effector T-cell development and antibody production and can increase vascular permeability
IL-1β (137, 138)	Activated macrophages, monocytes, and subset of dendritic cells; is proteolytically cleaved by caspase-1 into its biologically active form by the NLRP3 inflammasome	Mature IL-1β binds to IL-1R1, which undergoes a structural change allowing the coreceptor IL-1R3 to bind and form a trimeric complex	The intracellular TIR domains of each receptor recruit MyD88; downstream phosphorylation of IL1R associated kinases and inhibitor of NFκB kinaseβ.	IL1R is ubiquitously expressed and induces tissues to produce inflammatory cytokines such as IL-6 as well as chemokines and lipid mediators promoting edema such as Prostaglandin E2. It plays an important role in mediating neuroinflammation by inducing cyclooxygenase-2 (PGS2/COX2) in the CNS.
IL-18 (139)	Macrophages; like IL-1β, IL-18 is a pro-peptide cleaved into its biologically active form by the NLRP3 inflammasome	Binds to its receptor IL-18Rα, which induces recruitment of the constitutively expressed co-receptor IL-18Rβ	Signals through the toll/IL-1R (TIR) domain, recruiting the MyD88 adaptor protein and activating inflammatory programs and the NF-κB pathway.	IL-18 (along with IL-12 or IL-15). acts on T cells and NK-cells to induce IFNγ production generating an inflammatory feedback loop. The activity of IL-18 can be suppressed by its endogenous antagonist IL-18BP (constitutively expressed/secreted by mononuclear cells)

**Table 8:** Management considerations for suspected or confirmed HLH-disorders.

Management considerations for suspected or confirmed HLH-disorders	
**Management of HLH-disorders should begin as soon as the syndrome has been recognized. Assessment of bone marrow and CNS involvement should not delay therapy in critically ill patients. For patients with suspected malignancy, diagnosis should be established prior to initiation of steroids/chemotherapy. Principles of management are aimed at controlling the acute hyperinflammatory feedback loop (employing cytokine-modulating and immunosuppressive and chemotherapeutic agents as indicated) and providing meticulous supportive care until the underlying etiology can be effectively and safely addressed.** **HEMATOLOGY/ONCOLOGY:** - Serial monitoring of CBCs and blood product support to correct anemia and thrombocytopenia (generally Hgb >7g/dL, Platelets >10-50K/*μ*L depending on bleeding risk/CNS involvement) - Serial monitoring of coagulation studies/fibrinogen, correction of coagulopathy with FFP/cryoprecipitate (generally Fibrinogen >150, PT <15) and control of bleeding - Monitor therapeutic levels of calcineurin inhibitors if employed **CARDIOPULMONARY:** - Close vital sign monitoring - Ensure hemodynamic stability (consider echocardiogram), vasopressor support as necessary - Provide supplementary oxygen, non-invasive or protective invasive ventilation strategies as indicated **FEN/GI/ENDO:** - Serial monitoring of electrolytes, LFTs/bilis, blood gases. Manage electrolytes, control acid base disorders and fluid status - Provide appropriate nutrition (enteral vs. TPN) - Gastroprotection/PPI while on steroids - Serum glucose monitoring and management (especially while on steroids) **INFECTIOUS:** - Monitor IgG and replete with IVIG (generally >400) - PJP prophylaxis - Fungal prophylaxis (agent selection impacted by liver function) - Antibiotic prophylaxis in neutropenic (ANC <200), non-febrile patients - Empiric antibiotic therapy in febrile patients - Viral screening as indicated - HSV/VZV prophylaxis as indicated - RSV prophylaxis as indicated **NEUROLOGY:** - Serial monitoring and neurologic exams to evaluate for involvement or progression to CNS involvement - Management of seizures and seizure prophylaxis, as indicated, in consultation with Neurology **INFLAMMATION:** - Serial monitoring of CXCL9, IL-18, sIL2R (CD25), ferritin, CRP, ESR, triglycerides, cytokine panels (if available) **ACCESS:** - Ensure appropriate central access (PICC line, central venous catheter)	
Primary HLH	**Initial therapy (per HLH-2004):** - Dexamethasone 10mg/m2/d x 2 weeks, 5mg/m2/d x 2 weeks, 2.5mg/m2 x 2 weeks, 1.25mg/m^2^ x 1 week, taper and discontinue during 8^th^ week. Pulse with 10mg/m2/d x 3 days Q2 weeks during continuation therapy/until HCT - Etoposide 150mg/m2 IV twice weekly x 2 weeks, then weekly during initial therapy. Q2 weeks during continuation therapy +/- - IT therapy with -based methotrexate and hydrocortisone for patients with CNS involvement (start weekly IT therapy week 3 only if progressive neurologic symptoms or an abnormal CSF on standard evaluation at diagnosis and Week 2 has not improved) **Refractory to initial therapy, recurrent or progressive disease or unable to tolerate conventional HLH therapy:** - Emapalumab starting dose 1mg/kg IV Q3–4 days. Dose escalation based on response per FDA label (28). Continue glucocorticoids +/- Etoposide - Individualized approaches in consultation with Oncology and HCT (ruxolitinib, alemtuzumab, anakinra, tofacitinib, high dose IVIG) **Definitive therapy:** Allogeneic HCT during continuation therapy/once HLH controlled and donor available - Carefully taper IST towards HCT - Reduced-intensity conditioning regimens (e.g. alemtuzumab, melphalan, thiotepa, fludarabine) (140) - Pre/peri-HCT use of emapalumab may improve risk of mixed chimerism and graft failure after RIC-HLH (30)
Rheumatic*	**Initial therapy:** - Anakinra 2-4mg/kg/dose (max 100mg) IV/SQ Q12-24hrs, up to Q6hrs (127) - IVIG (1-2g/kg/dose) per discretion of primary team **PLUS** In absence of concern for infection or in critical patients (malignancy ruled out): Methylprednisolone 30mg/kg/d x 3 days (max 1000mg), followed by 1-2mg/kg/d divided Q12 **+/-** Calcineurin inhibitor, either: - Cyclosporine starting dose of enteral 3-7mg/kg/d divided Q12 or 1-2.5mg/g/d divided Q12 IV (goal trough: 50-100ng/mL) (127) **OR** - Tacrolimus enteral starting dose of 0.1mg/kg/d divided Q12 or 0.03mg/kg/d IV continuous infusion (goal trough: 5–15 ng/mL) **Refractory to initial therapy:** - Emapalumab 2x/wk (Day 1: 6mg/kg IV, Day 3: 3mg/kg/d. Continue 3mg/kg 2x/wk. If no response, can increase to 6mg/kd/d on day 3 and then 10mg/kg/d on day 9 **OR** - Ruxolitinib </= 10kg: 2.5mg PO BID, >10-20kg: 5mg PO BID, >20kg 10mg PO BID If no/incomplete response after 3 days increase up to 25mg/m2 BID with max dose 25mg BID **Definitive therapy:** Diagnose and manage underlying rheumatologic condition. Avoid MAS triggers. **For patients with recurrent episodes and/or current lung disease:** Consider allogeneic HCT
Infection	**Initial therapy:** Identify and treat underlying infection E.g. rituximab for EBV, antibiotics for bacterial infection **Definitive therapy**: Consideration of allogeneic HCT if underlying IEI
Malignancy	**M-HLH at presentation:** Diagnose the underlying malignancy - Initiate tumor specific chemotherapy if safely feasible - Corticosteroids (Prednisolone 1-2mg/kg/d or Dexamethasone 5-10mg/m2) - Anakinra 2-4mg/kg/dose (max 100mg) IV/SQ Q12-24hrs, up to Q6hrs Alternatives/Individualized approach: - Ruxolitinib - Etoposide - IVIG - Emapalumab **M-HLH during chemotherapy:** Consider holding chemotherapy Evaluate for infections, relapse, secondary malignancy Consider Corticosteroids (Prednisolone 1-2mg/kg/d or Dexamethasone 5-10mg/m2) **OR** Anakinra 2-4mg/kg/dose (max 100mg) IV/SQ Q12-24hrs, up to Q6hrs Alternatives/Individualized approach: - IVIG (2g/kg over 2–3 days) - Cautious use of etoposide
IEC	**CRS (** 109): - Supportive management - Tocilizumab (>/=30kg: 8mg/kg IV, <30kg: 12mg/kg IV. Max 800mg/dose) for Grade 2–3 or persistent fevers for Grade 1 (may redose Q8hrs, but limit to 3 doses/24hrs and strongly consider alternative therapies if unresponsive to tocilizumab after 1–2 doses) (115) - Addition of corticosteroids (dexamethasone 0.5mg/kg per dose [max 5-10mg] IV up to Q6hrs or 1-2mg/kg/d methylprednisolone) for Grade 3/progressive CRS. Close reassessment and wean as tolerated (104, 110). Escalation to Grade 4: Methylprednisolone 30mg/kg/d IV (max 1000mg) divided Q12hrs followed by rapid taper. Consider methylprednisolone 1000mg IV BID for refractory Grade 4 Alternative agents for refractory CRS: - Anakinra 2-10mg/kg/d (max 100mg/dose, max daily dose of 400mg) IV/SQ Q6-Q24hrs (111) - Emapalumab 1mg/kg IV x 1 (121) - Dasatinib (limited data) (141) - Siltuximab 11mg/kg IV once (142) - Cyclophosphamide - Antithymocyte globulin **ICANS:** - Corticosteroids. Consider for ICANS Grade 2, administer for ≤Grade 3: (Dexamethasone 1mg/kg/dose, max 10mg IV/dose Q6–12 hours with close reassessment and weaning as tolerated. Grade 4 ICANS: Methylprednisolone 30mg/kg/d IV (max 1000mg) QD or BID. - ICANS grade ≥1 with concurrent CRS: Administer tocilizumab at CRS dosing (109, 110) Refractory ICANS: - Anakinra 2-10mg/kg/d (max 100mg/dose, max daily dose of 400mg) IV/SQ Q6-Q24hrs (111). Continuous IV infusion can be considered - IT therapies (diagnostic and therapeutic LP with judicious drainage of CSF, +/- IT hydrocortisone +/- IT AraC and methotrexate based on age-based dosing) (143, 144) **IEC-HS:** First-line/mild-moderate: - Anakinra 2-10mg/kg/d (max daily dose of 400mg) IV/SQ Q6-Q24hrs or continuous infusion (111) +/- corticosteroids (Pediatrics: 10mg/m2/d IV or methylprednisolone 1-2mg/kg Q6–12 hours; Adults: dexamethasone 10mg-40mg/d, typically 10mg IV Q6hrs) (114) Second-line/moderate-severe: - Maximize anakinra dose if initiated at <10mg/kg/d dosing - Corticosteroids: (Pediatrics: 10mg/m2/d IV or Methylprednisolone 1-2mg/kg/d divided Q6–12 hours; Adults: dexamethasone 10mg-40mg/d, typically 10mg IV Q6hrs) (114) - Consider ruxolitinib (>/= 14yo: 10mg BID, option to increase to 20mg BID; <14yo: >25kg: 5mg BID; <25kg: 2.5mg BID) (114, 145) **OR** - Consider emapalumab (1mg/kg x 1 dose, repeat dosing Q3–4 days with option to increase dose up to 3-6mg/kg) (114, 145) Third-line/severe: - Emapalumab (consider initial dose at 6mg/kg IV and subsequent de-escalation at Q3–4 days dosing in patients with severe IEC-HS. Published dosing is per package insert: 1mg/kg x 1 dose, repeat dosing Q3–4 days with option to increase dose up to 3-6mg/kg) (121) **OR** - Ruxolitinib (>/= 14yo: 10mg BID, option to increase to 20mg BID; <14yo: >25kg: 5mg BID; <25kg: 2.5mg BID) (114, 145) - Consider etoposide (50-100mg/m2 x 1, both pediatric and adult) (114, 145)

AraC, cytarabine; IST, immune suppressive therapy; IT, intrathecal; SQ, subcutaneous.

Management recommendations may continue to change over time, and decisions about evaluation, diagnosis and/or treatment are the responsibility of the patient’s treating clinician and should be tailored to the individual patient’s clinical care needs. The medication dosing contained within this Table is provided as a reference only. Please refer to your institutional formulary or ordering guidelines when placing orders for the clinical care of patients. Additionally, infection prophylaxis is not detailed above and should be considered per local guidelines and clinician judgment.*Guidelines informed by our institutional EBG [described in text and in ref (55)], with modifications based on experience with additional agents since the initial publication of this EBG.

In patients undergoing IEC therapies, standard institutional algorithms should be employed to monitor for the evolution of HLH-like toxicities. While the diagnosis and management of CRS and ICANS is distinct from the more recently described entity of IEC-HS, there is substantial overlap in presentation, diagnostic parameters, and management. CRS typically occurs more proximal to the IEC infusion and is managed with tocilizumab as the first-line agent, with addition of corticosteroids for high-grade persistent or recurrent symptoms. Anakinra and emapalumab, amongst other agents, have been used for refractory cases. The management of ICANS focuses on the use of corticosteroids, anakinra in refractory cases, and the addition of intrathecal hydrocortisone for severe, refractory situations. In contrast, IEC-HS timing is distinct and presents during improvement in CRS or after CRS resolution. IFN-γ plays a key role, and in addition to anakinra and/or corticosteroids, ruxolitinib or emapalumab may be employed.

## Conclusion

In summary, HLH and HLH-like syndromes represent life-threatening entities in the pediatric setting and frequently require multidisciplinary critical care management with involvement by intensivists, rheumatology, immunology, oncology, infectious disease, and pediatric cell therapists. While the underlying etiology and the type and intensity of triggers required to produce the clinical HLH/HLH-like syndrome varies, these different entities ultimately converge to produce very similar clinical manifestations. Despite very distinct defects in the underlying immunologic pathophysiology, there is substantial overlap in the cytokines involved in the terminal pathways of the inflammatory feedback loop and the available therapeutics to mitigate the resulting toxicity. In this review, we aimed to outline differentiating features and common pathways and provide a framework for the management of these complex patients in the pediatric cell therapy and critical care setting.
